# Deliberate self-poisoning in children and adolescents

**DOI:** 10.1192/j.eurpsy.2024.1649

**Published:** 2024-08-27

**Authors:** S. Hmimou, S. Boukhorb, S. Irnat, F. Hadrya, N. Rhalem, M. A. Bellimam, A. Soulaymani, A. Mokhtari, R. Soulaymani-Bencheikh, H. Hami

**Affiliations:** ^1^Laboratory of Biology and Health, Faculty of Sciences, Ibn Tofail University, Kenitra; ^2^University Hassan First of Settat, Higher Institute of Health Sciences, Health Sciences and Technologies Laboratory, Settat; ^3^ Moroccan Poison Control Center; ^4^Forensic Institute of Royal Gendarmerie, Rabat, Morocco

## Abstract

**Introduction:**

Suicide is a significant global public health issue that has a severe impact on children and adolescents.

**Objectives:**

This study examined the epidemiological features of self-poisoning events among these groups in Morocco.

**Methods:**

In this retrospective study, data on intentional poisoning cases among children under 15 years of age and adolescents aged 15-19 years were analyzed. The Moroccan Poison Control Center has reported these cases over a period of 34 years.

**Results:**

During the study period, 7,111 deliberate self-poisoning cases were documented among children and adolescents, representing 30% of all reported self-poisoning cases (out of a total of 23,711 cases with known ages). The vast majority of the cases (80.8%) involved females, indicating a significant female-to-male ratio of 4.2. The patients had a mean age of 16.05 ± 2.10 years. Notably, drugs were the predominant method of self-poisoning, comprising 51.7% of the cases, followed by pesticides at 31.3%. The symptoms of poisoning manifested with significant variation, contingent on the type of toxin involved, the amount ingested, and the time passed before medical care was administered. Of the 4,711 cases with known outcomes, 144 (3.06%) were fatal. Nonetheless, the outcomes were favorable for the remaining cases, with or without lasting sequelae.

**Conclusions:**

The ongoing prevalence of suicide and suicide attempts among children and adolescents is a prominent issue in public health. Our research emphasizes the crucial necessity to address suicide, as it remains one of the primary causes of mortality in young individuals.

**Disclosure of Interest:**

None Declared

